# Revisiting food addiction in the era of GLP-1–based obesity pharmacotherapy via neural reward pathways linking feeding and substance use

**DOI:** 10.3389/fnbeh.2026.1805953

**Published:** 2026-04-13

**Authors:** Richard M. O’Connor

**Affiliations:** Nash Family Department of Neuroscience, Icahn School of Medicine, Mount Sinai Hospital, New York, NY, United States

**Keywords:** central nervous system, feeding behavior, GLP-1 receptor agonists, gut–brain axis, obesity

## Abstract

Obesity rates, once thought to be on an inevitable rise with limited treatment options, have recently stabilized and begun to decline in the US, a trend that coincides with the increased use of GLP-1 receptor agonist medications for weight management. Accumulating evidence indicates that GLP-1 receptor agonists influence feeding behavior through central neural pathways that also regulate compulsive and reward-driven actions. GLP-1 receptor agonists not only reduce appetite but also reshape food preferences, shedding light on the neural mechanisms underlying compulsive consumption and offering potential therapeutic avenues for metabolic and behavioral disorders. As such, GLP-1 receptor agonists have the potential to reinforce the concept of obesity as a treatable medical condition. As these pharmacotherapies reshape household grocery expenditures and inspire new food product lines tailored to users’ nutritional needs, they are catalyzing broader economic and social transformations. Furthermore, ongoing innovations in these therapies hold promise for expanded clinical applications and improved health outcomes. This review reconsiders the debated concept of ‘food addiction’ in light of these pharmacological advances, drawing on neurobiological, societal, and emerging clinical perspectives.

## 1 Introduction

Until recently, the worldwide increase in obesity, characterized by a body mass index (BMI) exceeding 30 kg/m^2^, was widely considered an inevitable trend with limited hope for meaningful advances in pharmacological treatment. In fact, between 1960 and 2010 the proportion of the US people considered to be obese doubled from 15 to 30% (Flegal et al., 2010; Gaziano, 2010). While obesity is often described as a simple imbalance in energy homeostasis, where calorie intake exceeds expenditure, this explanation fails to capture the true complexity of the disorder, which reflects a dynamic interplay of genetic and environmental factors that shape energy balance. When attempting to explain the dramatic rise in the levels of obesity one must consider the conspicuous changes coinciding in the food environment. Since the 1960s, the dollar cost per unit of energy in food has decreased dramatically for processed foods (Drewnowski, 2003), resulting in a 20% increase in the per capita availability of sugar and fat since 1977 (Drewnowski, 2003). Notably, there is a clear inverse relationship between the degree of food processing and the monetary cost per calorie, making highly processed foods, typically rich in fats and sugars, far more accessible to the general population (Ravandi et al., 2025). These macronutrients are widely recognized as the primary drivers of excessive food intake (Kenny, 2011a; O'Connor et al., 2015; Gearhardt and DiFeliceantonio, 2023; LaFata et al., 2025). A wealth of compelling studies have demonstrated that not only are the brain’s reward circuits in humans and rodents exceptionally sensitive to the composition of these foods (Stice et al., 2011; Stuber and Wise, 2016) but repeated exposure to such foods can profoundly reshape function of such brain circuits that further promote the intake of these calorie dense foods at the expense of less palatable, lower calorie foods that offer an overall superior nutritional profile (Wang et al., 2001; Fetissov et al., 2002; Geiger et al., 2008; Stice et al., 2008; Volkow et al., 2008; Johnson and Kenny, 2010; Stice et al., 2010a; Stice et al., 2010b; Matikainen-Ankney et al., 2020; van Galen et al., 2023; Stuber et al., 2025). This is considered at least partially responsible for the fact that most attempts to lose weight through dieting fail (Stalonas and Kirschenbaum, 1985; Wadden et al., 1989), with overweight individuals typically resuming food intake at levels that maintain a persistent state of positive energy balance. The persistent difficulty in regulating food intake has contributed to the increasing acceptance of uncontrolled eating qualifying as an addiction disorder, similar to established models of drug abuse. Compulsive use, marked by an inability to abstain or limit intake in spite of awareness of harmful outcomes, is a defining characteristic of substance use disorders (SUDs) (Health et al., 1988; O'Brien, 2005; DSM-V, 2013; Gearhardt and DiFeliceantonio, 2023; LaFata et al., 2025) and such a pattern of food-intake behavior closely mirrors the compulsive consumption observed in drug addiction, where people are unable to abstain or moderate usage despite recognizing the associated risks (DSM-V, 2013). Such individuals often continue consuming excessive amounts of food even when fully aware of the negative consequences, such as health problems, decreased mobility, and unwanted physical changes (Booth et al., 2008; Puhl et al., 2008; Neovius et al., 2009; Gaziano, 2010; Dietrich and Horvath, 2012; Borrell and Samuel, 2014). In contrast, hedonic eating refers to consuming food for its pleasurable taste and reward, rather than to meet metabolic needs (Lowe and Butryn, 2007), and does not necessarily imply loss of control or compulsive overeating. Accordingly, various conceptualizations of “food addiction” reflect the DSM criteria for SUDs, emphasizing loss of control as a central element (Volkow and Wise, 2005; Tanofsky-Kraff et al., 2008; Gearhardt et al., 2009a, 2009b; Gearhardt et al., 2011; Kenny, 2011a, 2011b; Collins et al., 2021; O'Connor and Kenny, 2022; Gearhardt and DiFeliceantonio, 2023; LaFata et al., 2025). Therefore, for any pharmacological or behavioral intervention to be considered effective in treating food addiction, it must help individuals restore their ability to regulate both the quantity and types of food they consume.

For decades, it seemed that rising population-level weight gain in the US was an unavoidable pattern; however, the average BMI plateaued in 2022 and declined in 2023 (Rader et al., 2024). Notably, this shift closely paralleled the expanded use of glucagon-like peptide-1 receptor agonist (GLP-1 RA) medications for weight management. Although it is important to recognize that, just as the rise in obesity has been driven by multiple interacting factors, its recent decline is also likely shaped by a complex interplay of causes. Importantly, the largest reductions in BMI were observed in the southern United States, which also reported the highest rates of GLP-1 RA dispensing claims (Rader et al., 2024), a geographic concordance that is consistent with a causal contribution of these pharmacotherapies to the observed population-level BMI decline.

The early success of these drugs in combating what was considered to be an intractable public health concern has begun to reshape how society is framing obesity as a health issue, leading to ripple effects that are affecting how food is sold and consumed, insurance and workplace wellness policies, and importantly, public attitudes surrounding the veracity of framing obesity as a medical issue that can and should be treated. In this review I aim to re-examine the contested concept of ‘food addiction’ as a disease in the context of emerging GLP-1–based obesity pharmacotherapies, integrating perspectives from neurobiology, societal impacts, and early evidence for their potential application in other substance-use disorders.

## 2 Mechanisms of action and ongoing therapeutic innovations in GLP-1 receptor agonists

All GLP-1 RAs mimic the endogenous hormone GLP-1, binding to GLP-1 receptors (GLP-1 Rs) distributed in pancreatic islets, the gastrointestinal tract, and the brain. Activation of GLP-1 Rs triggers glucose-dependent insulin secretion, suppression of glucagon release, delayed gastric emptying, and promotion of satiety (Kim and Egan, 2008; Zheng et al., 2024). In one of the first studies to provide exogenous GLP-1 to humans it was found plasma insulin levels rose significantly and glucose and glucagon concentrations fell (Kreymann et al., 1987). All GLP-1 RAs induce a significant reduction in energy intake, which is the primary driver of their weight loss efficacy. Clinical studies (in both obesity and type 2 diabetes) consistently show that GLP-1 RA treatment leads to less hunger, increased satiety, and spontaneous decreases in daily caloric consumption (Zheng et al., 2024). In rodent models, peripheral administration of exendin-4 (EX4), a GLP-1RA has been shown to reduce motivation for sucrose, as evidenced by decreased responding in progressive ratio operant-conditioning tasks (Dickson et al., 2012). Furthermore, targeted delivery of exendin-4 to the nucleus of the solitary tract (NTS) similarly diminished both operant responding for sucrose under a progressive ratio schedule and conditioned place preference for food rewards in rats (Richard et al., 2015). These findings collectively underscore the capacity of GLP-1R agonism to modulate reward-driven feeding behaviors.

Currently available FDA-approved GLP-1 RAs include several monotherapy agents and one combination multi-agonist, the dual GLP-1/GIP agonist tirzepatide (Zheng et al., 2024). Across all these drugs, the common mechanism of action are glucose lowering via *β*-cell GLP-1R, slowed gastric emptying (gut) and appetite suppression via central effects (McLean et al., 2021). Therapeutic benefits to the cardiovascular and renal systems are emerging yet it currently remains unresolved if these are via direct actions on GLP-1R on these tissues or an indirect effect mediated by the health benefits of weight loss.

Exenatide was the world’s first clinically used GLP-1RA, developed in 1995 and approved for the treatment of type 2 diabetes mellitus (T2DM) in 2005 due to its proven ability to regulate postprandial glucose levels effectively (DeFronzo et al., 2005; Bunck et al., 2009; Furman, 2012). While exenatide provides clinically meaningful glycemic control, its impact on weight loss is relatively modest compared to more recent GLP-1R agents, resulting in less than 3 kg of weight reduction over a 30-week period (DeFronzo et al., 2005; Bunck et al., 2009). Furthermore, its short half-life of less than 3 h (Min et al., 2025) necessitates twice-daily injections to maintain efficacy, which can be a limiting factor for long-term patient adherence.

Lixisenatide, a structurally modified derivative of exendin-4, addresses some of these limitations by offering a longer half-life of approximately 3 h, thereby permitting once-daily dosing (Leon et al., 2017). Like exenatide, lixisenatide demonstrates reliable control of blood glucose and provides modest weight loss, typically less than 3 kg depending on the duration of treatment (Sheng et al., 2024).

Building on these advances, liraglutide represented a significant step forward as a synthetic human GLP-1RA approved for both T2DM (Tamborlane et al., 2019) and obesity (Feinglos et al., 2005; O'Neil et al., 2018). Liraglutide’s mechanism involves appetite suppression, which leads to reduced energy intake and clinically relevant weight loss (Feinglos et al., 2005; Pi-Sunyer et al., 2015; Min et al., 2025). Its molecular design includes a fatty acid chain that enables albumin binding, resulting in slower absorption and increased resistance to degradation. This confers a half-life of around 12 h, providing 24-h glycemic control with a single daily injection (Jackson et al., 2010).

Dulaglutide, a modified analog of human GLP-1 covalently linked to IgG4, possesses a notably extended half-life of about 90 h, thereby expanding its therapeutic applications. Dulaglutide is approved for T2DM management (Sanford, 2014), offering glycemic control and weight loss benefits comparable to liraglutide but with the added convenience of a weekly dosing regimen (Chang et al., 2020).

Semaglutide, another modified analog of human GLP-1, is resistant to DPP-4 degradation, which enables a once-weekly injection (Lau et al., 2015). Semaglutide not only consistently reduces energy intake (Blundell et al., 2017) but also achieves substantial weight loss, up to approximately 15% in individuals who are overweight or obese (Wilding et al., 2021). The enhanced potency and extended dosing interval of semaglutide mark a significant advancement in GLP-1 RA therapy for both glycemic and weight management.

The introduction of tirzepatide, the first dual agonist targeting both the GLP-1R and the glucose-dependent insulinotropic polypeptide (GIP) receptor, represents a new frontier in incretin-based therapy (Syed, 2022; Nogueiras et al., 2023). Tirzepatide has demonstrated superior efficacy in both weight loss and blood glucose control compared to semaglutide (Aronne et al., 2025), highlighting the therapeutic potential of multi-agonist strategies.

Collectively, the progression from exenatide to tirzepatide illustrates how extending the half-life of GLP-1R agents and incorporating additional mechanisms of action has led to substantial improvements in both glycemic control and weight loss. These innovations suggest that as researchers continue to refine and expand the pharmacological profiles of GLP-1RAs, their efficacy across multiple therapeutic domains will likely continue to increase, paving the way for broader applications and improved patient outcomes.

## 3 GLP-1R localization and mechanistic pathways in metabolic and behavioral regulation

**Figure 1:**
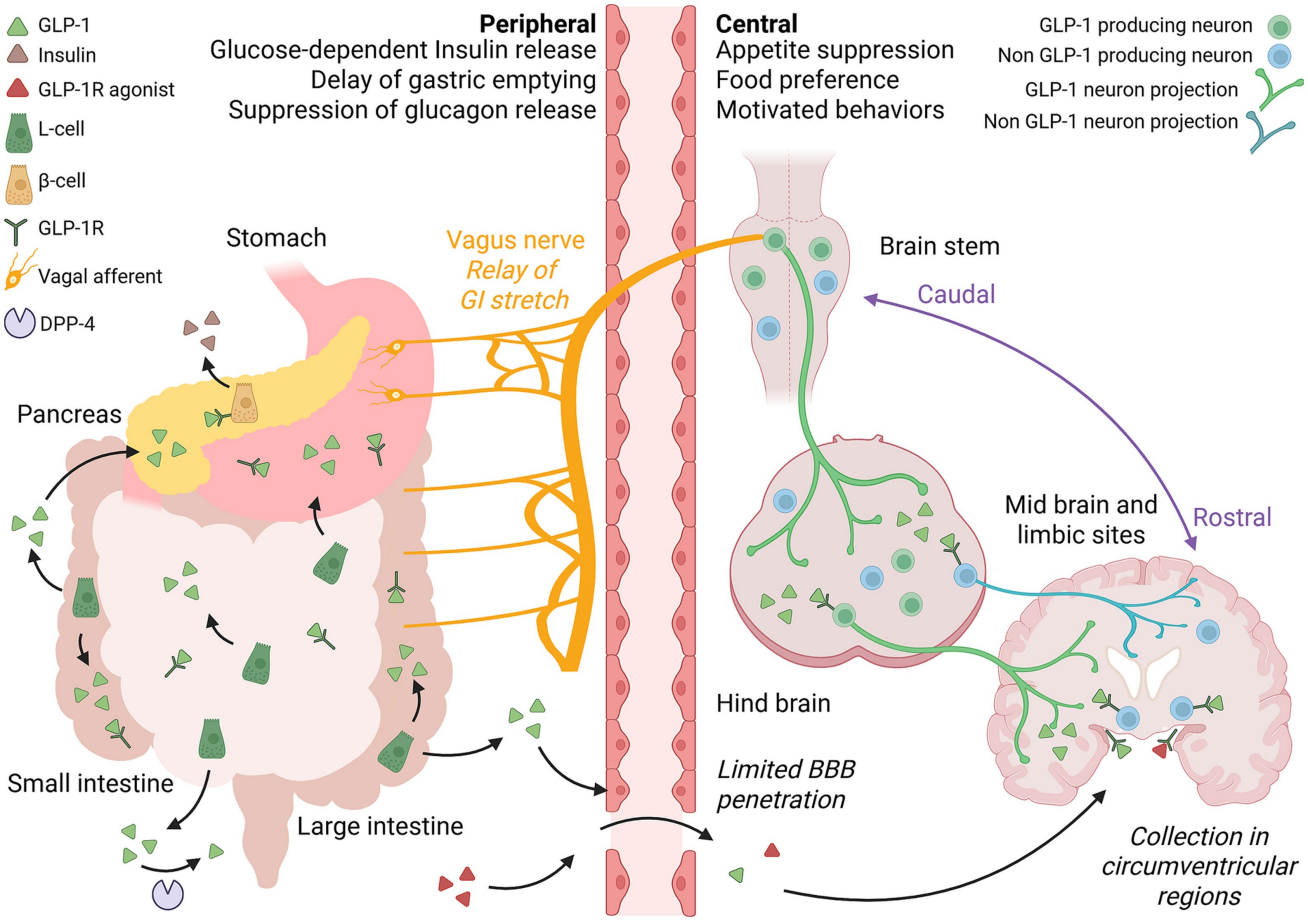
Localization and mechanistic pathways underlying the metabolic and appetite-regulating actions of GLP-1 and its receptor agonists.

GLP-1 Rs exhibit high expression in pancreatic *β*-cells in both rodents and humans, where they play a pivotal role in mediating glucose-dependent insulin secretion (Nauck et al., 1993; Montrose-Rafizadeh et al., 1994; Tornehave et al., 2008) (Figure 1). This fundamental action anchors the glucose-lowering efficacy of GLP-1 RAs and establishes their therapeutic relevance in diabetes management. Beyond the pancreas, GLP-1Rs are also present in the gastric mucosa of the human stomach (Broide et al., 2013) and within enteric neurons distributed throughout the intestine (Richards et al., 2014; Drokhlyansky et al., 2020). Signaling via pancreatic β-cells underlies the insulinotropic effects of GLP-1 RAs, while activation of enteric GLP-1Rs delays gastric emptying, effectively blunting postprandial glucose spikes and prolonging satiety (Kreymann et al., 1987; Nauck et al., 1993; Zheng et al., 2024).

GLP-1 RAs also gain access to hindbrain sites indirectly via the vagus nerve. Notably, ablation of vagal inputs, whether by surgical or chemical methods, has been shown to block the appetite-suppressing effects of peripheral GLP-1 in rodent models (Abbott et al., 2005; Kanoski et al., 2011; Labouesse et al., 2012; Brierley and de Lartigue, 2022), underscoring the essential role of these neural connections in relaying gastrointestinal signals to the central nervous system. GLP-1 Rs are abundantly expressed on stomach-innervating vagal afferent neurons which are critical for detecting gastrointestinal stretch and transmitting this information to the nucleus tractus solitarius (NTS; Bai et al., 2019; Williams et al., 2025). In line with this, the gastric emptying delay induced by GLP-1RAs is dependent on the presence of GLP-1Rs on these vagal afferents (Imeryuz et al., 1997). Further supporting this mechanism, peripheral GLP-1 infusion improves glucose tolerance in intact animals, but this benefit is lost following the ablation of vagal signaling (Imeryuz et al., 1997; Abbott et al., 2005). This finding is mirrored in human studies, where exogenous GLP-1 fails to enhance insulin secretion or reduce postprandial glucose levels in individuals who have undergone vagotomy (Plamboeck et al., 2013). Collectively, these results highlight the role of vagal afferents in mediating the metabolic and appetite-regulating effects of peripheral GLP-1, thereby linking gut-derived signals to central regulatory circuits.

In addition to their peripheral actions, GLP-1Rs are also expressed throughout the central nervous system, particularly within hypothalamic nuclei, septal regions, the brainstem and at lower levels in various cortical structures (Pyke et al., 2014; Richards et al., 2014; Cork et al., 2015; Harasta et al., 2015; Heppner et al., 2015; Farr et al., 2016; Csajbok et al., 2019; Graham et al., 2020; Dossat et al., 2023). At these locations, GLP-1 RAs are believed to exert their appetite-suppressing effects, linking peripheral metabolic signaling with central regulation of feeding behavior. However, recent insights suggest that the gut and brain GLP-1 systems operate largely independently with mRNA transcripts for proglucagon (GCG), the precursor peptide for GLP-1, being abundant in the brainstem, particularly within the nucleus tractus solitarius (NTS; Drucker and Asa, 1988; Merchenthaler et al., 1999; Holt et al., 2019). GLP-1-producing neurons in the NTS project axon terminals widely, densely innervating regions involved in autonomic and metabolic control, such as hypothalamic and thalamic nuclei and the NTS itself (Larsen et al., 1997; Llewellyn-Smith et al., 2011; Ong et al., 2017; Tuesta et al., 2017). Furthermore, compelling evidence implicates this local, brainstem-derived GLP-1, rather than gut-derived GLP-1, as a principal modulator of physiological GLP-1R dependent signals that regulate appetite and body weight (Drucker and Asa, 1988; Merchenthaler et al., 1999). Circulating GLP-1 exists only at low picomolar concentrations, is rapidly inactivated by DPP-4 enzymes and is swiftly cleared by the kidneys (Hansen et al., 1999; Mulvihill and Drucker, 2014). This rapid turnover makes it unlikely that peripheral GLP-1 significantly contributes to endogenous activation of central GLP-1Rs. Furthermore, the degree to which various exogenous GLP1-RAs penetrate the blood–brain barrier (BBB) and access central receptors remains unresolved and an ongoing area of research. For example, studies using radiolabeled exenatide have shown that it can readily enter the mouse brain, though this central uptake appears to diminish at higher doses (Kastin and Akerstrom, 2003). In contrast, the longer-acting GLP-1RAs such as liraglutide and semaglutide, engineered for greater resistance to degradation, demonstrate poor penetration into the central nervous system (Salameh et al., 2020). This suggests that the chemical modifications enhancing their stability may simultaneously limit their access to central GLP-1Rs. Similarly, tirzepatide, a dual GLP-1R and GIP receptor agonist, does not seem to cross the BBB efficiently (Rhea et al., 2024). In line with these observations, recent findings indicate that the appetite-suppressing actions of GLP-1RAs may also be mediated through receptors located in circumventricular organs outside the BBB. These specialized regions relay peripheral signals into deeper brain areas without direct engagement of preproglucagon (PPG) neurons, which constitute the brain’s endogenous GLP-1 source (McLean et al., 2021). For example, rodent studies reveal that labeled semaglutide accumulates predominantly in brainstem, septal nucleus, and hypothalamus, areas with more permissive BBB permeability (Gabery et al., 2020). Similarly, the appetite-suppressing effects of liraglutide have been traced to its action within the arcuate nucleus (ARC) of the hypothalamus, a region also characterized by a relatively permeable BBB (Secher et al., 2014). Taken together, these pharmacokinetic findings indicate that the centrally mediated effects of GLP-1RAs are likely due to limited brain penetration or action at sites adjacent to the BBB, rather than widespread distribution throughout the central nervous system. Instead, neuronal GLP-1Rs located outside the BBB primarily serve as peripheral targets for gut-derived or pharmacologically administered GLP-1 (Varin et al., 2019; McLean et al., 2021).

The neurobiological mechanisms underlying the benefits of combined GLP-1R and GIPR agonism achieved from compounds like tirzepatide remains to be fully delineated. However, current evidence points to at least partial mediation through central mechanisms as direct activation of central GIPRs can suppress food intake and decrease body weight, whereas deletion of these receptors blunts such effects and protects against obesity by altering feeding behavior (NamKoong et al., 2017; Zhang et al., 2021).

Beyond these principal sites of GLP-1R action, lower-level receptor expression has been documented in several additional tissues, including the heart, kidney, lungs, and testis (Pyke et al., 2014; Richards et al., 2014; Caltabiano et al., 2020). The physiological or therapeutic significance of GLP-1 RA engagement at these peripheral sites remains unclear and warrants further investigation.

Taken together, growing evidence demonstrates that the motivational effects of GLP-1RAs on feeding behavior are driven by central neural pathways. Notably, these circuits substantially overlap with those involved in the compulsive and reinforcing aspects of drug abuse. Thus, the technical advances in understanding the distribution and functional roles of GLP-1R across peripheral and central systems continue to inform the development of more effective therapies targeting both metabolic and behavioral domains.

## 4 Central GLP-1R pathways linking metabolic regulation and addiction circuits

GLP-1 Rs are broadly distributed in brain circuits that play pivotal roles in motivation, including the striatum, hypothalamus, ventral tegmental area (VTA), and extended limbic regions such as the central amygdala and the infralimbic/prelimbic cortex (Pyke et al., 2014; Richards et al., 2014; Heppner et al., 2015). Notably, GLP-1-producing neurons within the nucleus tractus solitarius (NTS) project axons to the VTA and other limbic structures, further embedding GLP-1 signaling within neural networks that regulate reward and motivation (Llewellyn-Smith et al., 2011; Merkel et al., 2025). This extensive anatomical integration results in substantial overlap between GLP-1 signaling pathways and the mesolimbic dopamine system, which is central to reward-related behaviors. Given this convergence, it is unsurprising that GLP-1Rs also contribute to the motivation to seek drugs of abuse. For example, GLP-1R agonism has been shown to reduce cocaine-seeking behaviors in mice, as measured by intravenous self-administration (IVSA) paradigms (Sorensen et al., 2015). Similarly, agents such as Ex-4 and other GLP-1RAs suppress both amphetamine- and cocaine-induced conditioned place preference (Egecioglu et al., 2013a; Graham et al., 2013). Furthermore, GLP-1R agonism has been found to reduce alcohol intake in mice (Egecioglu et al., 2013b; Colvin et al., 2020), and to disrupt the normal activity of dopamine neurons during drug engagement (Egecioglu et al., 2013a; Egecioglu et al., 2013b; Sorensen et al., 2015; Merkel et al., 2025). Semaglutide has demonstrated the capacity to decrease both recurrent alcohol consumption and total alcohol intake in rats by over 50% (Aranas et al., 2023). Additionally, intracerebroventricular delivery of Ex-4 has been found to dose-dependently attenuate cue-evoked dopamine activity, further underscoring the direct influence of GLP-1R signaling on neural circuits governing reinforcement and motivation (Konanur et al., 2020).

Collectively, the preclinical literature demonstrates that GLP-1RAs attenuate phasic dopamine responses and diminish the reinforcing properties of addictive substances. However, GLP-1R expression and action extend beyond these regions to include the brainstem, hypothalamic nuclei, and the amygdala (Larsen et al., 1997; Llewellyn-Smith et al., 2011; Pyke et al., 2014; Heppner et al., 2015). This broad central distribution allows GLP-1RAs to modulate additional neural pathways implicated in the motivational aspects of substance use. For instance, Ex-4 injections into the lateral hypothalamus (LH) and dorsomedial hippocampus have been shown to suppress ethanol intake (Colvin et al., 2020). Furthermore, targeted knockdown of GLP-1 Rs in the medial habenula (MHb) or pharmacological antagonism in the interpeduncular nucleus (IPN) increases nicotine consumption and reinstates preference for nicotine-paired environments (Tuesta et al., 2017). Conversely, infusion of Ex-4 into the MHb reduces nicotine intake (Duncan et al., 2019).

Translational studies in humans, though less extensive, lend support to the central role of GLP-1RAs in modulating reward circuitry. Treatment with exenatide has been shown to decrease food intake and attenuate food-related neural responses in key regions such as the insula, amygdala, putamen, and orbitofrontal cortex (van Bloemendaal et al., 2014). In clinical trials, exenatide did not significantly reduce heavy drinking days compared to placebo, but it did blunt fMRI alcohol cue reactivity in the ventral striatum and septal area, critical nodes for drug reward and addiction. Additionally, the exenatide treated group exhibited lower dopamine transporter availability relative to placebo, and exploratory analyses indicated that exenatide significantly reduced both heavy drinking days and total alcohol intake among obese patients (Klausen et al., 2022).

In summary, convergent evidence from both preclinical and clinical research underscores the capacity of GLP-1RAs to interact with neural circuits that govern reinforcement and motivation, networks that are also targeted by drugs of abuse. Although originally developed for the treatment of metabolic diseases, GLP-1-based therapies are now recognized for their influence on the same brain systems mediating drug-seeking and reward, suggesting therapeutic potential beyond glycemic control.

## 5 GLP-1RAs influence addiction-related criteria in food consumption behaviors

The Diagnostic and Statistical Manual of Mental Disorders (DSM-5) defines substance use disorders (SUDs) as conditions arising from maladaptive patterns of substance use across a range of drug classes, including alcohol, cannabis, opioids, stimulants, sedatives, hallucinogens, inhalants, and tobacco (DSM-V, 2013). Although DSM-5 diagnosis is defined through multiple behavioral criteria encompassing impaired control, social dysfunction, continued use despite harm, and tolerance or withdrawal, converging clinical and preclinical evidence indicates that the core pathological feature of SUDs is the progressive loss of control over intake (O'Brien, 2005). Although neither the DSM nor the International Classification of Diseases (ICD) formally recognizes addiction to highly palatable foods as a clinical diagnosis (LaFata et al., 2025), there is nevertheless a notable parallel between substance use disorders and certain patterns of eating behavior. Specifically, the progressive loss of behavioral control, manifested by persistent overconsumption despite adverse consequences, mirrors the sustained positive energy balance that underlies and perpetuates weight gain (O'Connor and Kenny, 2022; Gearhardt and DiFeliceantonio, 2023). This convergence between substance-related and eating-related compulsivity has prompted the use of specific aspects of food preference and intake as proxies for compulsive behaviors in the context of metabolic disease. Emerging evidence indicates GLP-1RA therapies not only influence overall appetite and satiety but also fundamentally alter food preference. By shifting dietary choices away from energy-dense, highly palatable foods toward more nutritious alternatives, GLP-1 RAs offer unique insight into the neural and behavioral mechanisms underlying compulsive consumption.

## 6 GLP-1RAs reduce the appeal of high-calorie foods

Increasing evidence suggests GLP-1RA-based medications do more than merely increase satiety but also may reconfigure feeding and preference behaviors in both humans and rodents. This is characterized by a shift in preference toward lower-calorie, less palatable foods, which are typically considered less desirable compared to calorie-dense alternatives (Blundell et al., 2017). Thus, reductions in energy intake with GLP-1R–based pharmacotherapies reflect not only diminished appetite but may also involve a concomitant shift in food preference toward less energy-dense foods. For example, GLP-1 analogs have been shown to diminish the desire for sweet, salty, fatty, and savory foods, further supporting their role in shifting food preferences toward less palatable options (Kadouh et al., 2020). Furthermore, in a 12-week trial, semaglutide was shown to reduce body weight predominantly through appetite suppression, but this effect was also accompanied by a marked reduction in preference for high-fat foods (Blundell et al., 2017). Participants treated with semaglutide experienced significantly diminished cravings for sweet, savory, and dairy-rich foods compared to those receiving placebo after 20 weeks of therapy and during the active phase of weight loss (Friedrichsen et al., 2021). Similarly, another study found that semaglutide treatment resulted in approximately a 39% decrease in calories consumed from high-fat, high-sugar items (Gibbons et al., 2021). Notably, the extent of weight loss achieved with GLP-1 RA therapy may be directly linked to the degree of reduction in preference for high-calorie foods as semaglutide was associated with reduced cravings for both savory and sugary foods, with decreases in craving scores correlating with greater weight loss (Blundell et al., 2017; Wharton et al., 2023) which persisted for up to 2 years (Wharton et al., 2023). This reduction in desire for highly palatable food items further underscores the capacity of GLP-1 RA-based interventions to not only suppress appetite but also to selectively modulate food preferences, contributing to sustained dietary changes and weight management.

Liraglutide has also been shown to significantly reduce overall energy intake and the consumption of all macronutrients, including carbohydrates, protein, and fat during controlled buffet settings (Quast et al., 2021). Furthermore, patients treated with the dual GLP-1R and GIP agonist tirzepatide exhibited lower Food Craving Inventory scores for sweets and high-fat “fast foods” compared to those receiving liraglutide, indicating a greater reduction in desire for these items during weight loss (Martin et al., 2025).

Beyond general food preferences, GLP-1 RA therapy also appears to modulate sensory responses. In a 16-week trial, liraglutide was found to reduce the expressed desire for palatable taste properties, including sweet, fatty, salty, and savory flavors in individuals with obesity (Kadouh et al., 2020). While there is limited evidence of direct impairment in taste perception, one trial did observe that individuals taking GLP-1 RAs scored lower on both taste and smell assessments (Waterless Empirical Taste Test (WETT®) and University of Pennsylvania Smell Identification Test (UPSIT®), respectively; Khan and Doty, 2025). Supporting these clinical findings, consumer data reveal that individuals taking GLP-1 RAs do not simply reduce spending on all food items equally. Instead, their decreased food purchases are disproportionately concentrated in processed foods and sugar-sweetened beverages, further underscoring the shift in food preference induced by GLP-1R-based treatments (Dilley et al., 2025).

Preclinical studies in rodents generally mirror the findings in humans. In one of the earliest studies investigating these changes, chronic liraglutide treatment in rats with ad libitum access to both standard chow and sweet candies led to a reduction in total calorie intake. Crucially, this decrease was primarily attributable to a reduction in candy consumption, while chow intake increased compared to vehicle-treated controls with access to both food types (Raun et al., 2007). Ex-4 specifically reduced licking behavior for both sucrose and lipid solutions, but did not alter licking for water, indicating its selective impact on the consumption of caloric liquids (Treesukosol and Moran, 2022). Interestingly, direct injections of Ex-4 into the gustatory cortex of mice maintained on a high-fat diet reduced intake only when the fat density of the test diet differed from that of the maintenance diet, regardless of whether the shift was from high- to low-fat or low- to high-fat relative to the maintenance diet. This suggests that gustatory cortex GLP-1R signaling may modulate the perception of palatability, particularly when foods differ in macronutrient composition from familiar diets (Dossat et al., 2023).

Similarly, the dual GLP-1R and GIP agonist tirzepatide promoted the intake of standard rodent chow at the expense of a high-fat diet in mice, suggesting a preferential shift toward less energy-dense foods (Geisler et al., 2023). Interestingly, the same study found that, in rats, tirzepatide suppressed lipid intake without affecting sucrose (carbohydrate) consumption in a choice-based assay, implying that the effects of these medications may be macronutrient specific (Geisler et al., 2023). Additional research demonstrated that both liraglutide and sibutramine (a serotonin-norepinephrine reuptake inhibitor) reduced food intake and weight gain in rats provided with chow and a high-fat, high-sugar diet. However, liraglutide achieved this primarily through a pronounced reduction in palatable food intake, whereas sibutramine reduced consumption of both chow and palatable foods (Hansen et al., 2012). When the “effort” required by rodents to obtain palatable food was assessed, exendin-4 was found to decrease operant responding on both fixed and progressive ratio schedules, further supporting a reduction in the motivational drive for palatable foods (Bernosky-Smith et al., 2016).

However, it is worth noting that most preclinical studies do not incorporate a dose titration phase, a gradual increase to the therapeutic maintenance dose, which is standard in clinical settings. Interestingly, a study that did implement this titration approach found during the maintenance phase, rats treated with semaglutide showed a unexpected marked increase in the intake of lower concentrations of sucrose compared to vehicle treated controls, despite continuing to consume chow at only 90% the level of vehicle-treated animals (Cawthon et al., 2023). Whether this finding can be replicated in future studies, and what significance it holds for the broader understanding of GLP-1R agonism’s effects on food preference, remains to be determined.

In line with clinical data, GLP-1R agonism may also directly influence food preference through modulation of taste receptor expression. For example, a high-fat diet increased the expression of the T1R3 taste receptor in rats, an effect that was reversed by chronic exendin-4 treatment. This intervention was accompanied by a decrease in preference for low-dose sucrose solutions (0.1 M) (Zhang et al., 2013). Notably, similar phenomena are observed with bariatric surgery, which also leads to reductions in sweet and fat preference, a process in which GLP-1 is believed to play a mediating role, as demonstrated in rodent models (le Roux et al., 2011; Mathes et al., 2016).

Interestingly, emerging evidence suggests that GLP-1RAs may also provide therapeutic behavioral modification beyond their effects on appetite and food preferences. For example, GLP-1R agonism appears to facilitate new learning, potentially by modifying neural circuits involved in reward and adaptive behavior (Hanssen et al., 2023). Individuals with impaired insulin sensitivity have been shown to experience diminished associative learning; however, treatment with liraglutide was able to rescue some of this impairment, indicating that GLP-1R agonism may help restore learning capacity (Hanssen et al., 2023). Although the significance of this finding requires further investigation, it suggests that GLP-1 RA therapy could help facilitate more adaptive behaviors, especially in individuals struggling with compulsive eating or substance use, by potentially enhancing their capacity for new learning and behavioral flexibility.

In summary GLP1-RA treatment consistently reduces preferences for high-fat, high-sugar foods, while showing increased consumption of low-fat and/or low sugar items.

## 7 The evolving public perception of obesity and food addiction

As the success of GLP-1RAs in treating overweight and obesity rises, it prompts reflection on how these pharmacological advances could reshape both public and professional perspectives. Increasingly, obesity may be seen less as a personal failing and more as a medical condition, like type 2 diabetes or high cholesterol, warranting comprehensive treatment. Recent survey data highlight the ongoing divide in public and professional attitudes toward obesity. For example, a 2023 study in the UK found that 57% of adults attributed being overweight primarily to a “lack of willpower” (Beeken and Wardle, 2013). Meanwhile, a comparable study in the United States showed that around 60% of participants supported the idea of classifying obesity as a disease (Puhl and Liu, 2015). Adding another layer of complexity, a multinational survey revealed that only 26% of clinicians viewed obesity as a chronic disease, and fewer than half routinely recorded it as such in patients’ medical files (Dixon et al., 2025). Counter to this attitude was the emergence of the body positivity movement in the 2010s that sought to promote self-acceptance, celebrate body shape diversity, and prioritize metabolic health, challenging the use of BMI or weight as sole indicators of health without considering the broader context (Gailey, 2022; Griffin et al., 2022; Holi, 2025). This movement emphasizes that obesity should not be viewed as a pathology requiring a cure (Holi, 2025), nor as an “epidemic” necessitating personal responsibility for its resolution (Gailey, 2022). Although never fully embraced by the medical community (Phelan et al., 2015; Stewart and Ogden, 2019) and, compelling evidence indicates that maintaining an elevated BMI over the long term significantly increases the risk of developing chronic diseases (Sainsbury and Hay, 2014), body positivity became a robust cultural narrative, encouraging society to respect the dignity of all body sizes and to question the assumption that thinness equates to health (Holi, 2025; Neratzi et al., 2026).

The introduction of highly efficacious and generally safe medications for obesity treatment, such as GLP-1RAs, has disrupted this framework, reframing obesity as a medical issue that can be treated pharmacologically. Notably, research indicates that individuals most interested in trying GLP-1 medications, and willing to accept common side effects, are those who report greater body shame, body surveillance, weight concerns, anti-fat bias, disordered eating behaviors, and higher BMIs, alongside lower body appreciation and body neutrality (Markey et al., 2025). Additionally, a multi-country study found substantial demand for GLP-1 medications among people without medical indications, highlighting the evolving landscape of body image and obesity treatment (Jensen et al., 2025).

Furthermore, the growing medicalization of obesity has perhaps, somewhat paradoxically, led to the emergence of new forms of weight-related stigma. Critics of GLP-1 therapies often portray those who use these medications as opting for an “easy way out” to achieve thinness, perpetuating longstanding biases like those historically associated with bariatric surgery (Heitmann, 2025). Research demonstrates that women who lose weight through anti-obesity medications are frequently judged more harshly and perceived as having taken a “shortcut” to weight loss (Post and Persky, 2024). Despite this, recent studies using large language models to analyze public sentiment suggest that most opinions about GLP-1R-based weight loss treatments are either positive or neutral (Somani et al., 2024). Reflecting a growing acceptance of obesity as a legitimate medical condition, at least in the United State, a 2024 employer health benefits survey found that 61% of respondents support expanding Medicare (federal health insurance program primarily for individuals aged 65 and older) coverage beyond treatment for T2DM to include GLP-1 therapies for weight management in overweight individuals (Kaiser Family, F, 2024).

Public discourse surrounding GLP-1RAs thus remains dynamic and unsettled. While some view these medications as transformative advances that legitimize obesity as a treatable disease, others worry that their increasing popularity could undermine longstanding efforts to deconstruct diet culture and promote body positivity.

## 8 Societal changes driven by uptake of GLP-1 weight loss medications

The sweeping changes in food preference and appetite brought on by GLP-1RA pharmacotherapies are producing extensive downstream effects that reach beyond the individual, shaping broader cultural, social, and economic landscapes. As these medications reduce overall energy intake, individuals are consuming fewer calories with estimates placing this reduction between 16 and 39%, or about 700 calories per day on average (Christensen et al., 2024). Notably, while comprehensive, peer-reviewed studies are still limited, early non-peer-reviewed survey data suggest a significant reduction in grocery expenditures, with households using GLP-1 medications spending approximately 5% less compared to similar non-user households (Hristakeva et al., 2024). Marketing analyses estimate this shift could cut $6.5 billion from US grocery spending, a figure poised to grow as the adoption of these drugs continues to rise (Hristakeva et al., 2024).

These economic changes are closely tied to evolving dietary patterns. In line with laboratory-controlled studies demonstrating altered food preferences, the reduction in food intake among GLP-1 users is disproportionately associated with decreased consumption of processed foods, while intake of fruits and vegetables has increased overall. The net result is a measurable shift in appetites and diet patterns resulting in less processed food, and greater consumption of nutritionally dense, unprocessed items (Dilley et al., 2025).

This pronounced transformation in dietary preferences and spending behaviors is prompting food manufacturers to shift their marketing strategy and adapt their product offerings. For example, in 2024, global food juggernaut Nestlé responded to this emerging trend by launching a “GLP-1-friendly” frozen food line.^1^ These products feature portion-controlled options, such as grain bowls, mini flatbread pizzas with cauliflower crust, and high-protein sandwich melts designed to support the nutritional needs of individuals consuming smaller portions due to medication-induced appetite suppression. Importantly, these meals are not marketed as ultra–low-calorie diet products; instead, their focus is on delivering high protein, fiber, and essential vitamins to help those on GLP-1 therapy maintain adequate nutrition within reduced portion sizes.

The ripple effect extends further into the food service sector. In late 2024, Smoothie King introduced a new menu of protein-dense smoothies, described as “small meal replacements with extra hydration and fiber for those on weight-loss injections”.^2^ This reflects a broader industry shift toward catering to the needs of individuals managing their weight with GLP-1 medications, emphasizing nutrient density and portion control.

## 9 Challenges and limitations to compliance with GLP-1 RA therapy

While GLP-1 RA therapies are highly effective for glycemic control and weight loss, real-world data indicate that many patients either discontinue or struggle to adhere to these treatments. Discontinuation rates within the first year are consistently reported between approximately 45 and 75% (Sikirica et al., 2017; Rodriguez et al., 2025), with those without T2DM exhibiting even higher rates of discontinuation than those with T2DM (Rodriguez et al., 2025). Specifically, first-year discontinuation rates are about 45% for patients with T2DM and 65% for those without (Rodriguez et al., 2025). When cost is excluded as a factor, the leading reason for discontinuation is gastrointestinal side effects, most notably nausea and vomiting, as reported by patients (Sikirica et al., 2017). Interestingly, the neural circuits responsible for the metabolic effects of GLP-1R agonism may be distinct from those mediating nausea or malaise (Huang et al., 2024), suggesting that future therapies could be tailored to preserve metabolic benefits while minimizing gastrointestinal side effects.

Dosing schedule and route have also been identified a major factor affecting compliance with a single weekly injection achieving just over 10% lower risk of discontinuation (Weeda et al., 2021). This is likely to become a less salient issue as drug developers develop new formulations with more convenient dosing routes and schedules since overall simpler dosing (fewer steps, less frequent dosing) tends to improve medication satisfaction and adherence (Boye et al., 2021). Furthermore, access to GLP1-RAs is a major issue for uptake remaining relatively low despite the high efficacy of these medications (Rodriguez et al., 2025). Cost remains a barrier with a person’s income being a major factor in determining compliance with GLP1-RAs (Rodriguez et al., 2025).

Furthermore, as of writing, in the Unites States, Medicaid which provides free or low-cost health coverage to low-income individuals is barred for providing access to GLP-1RA medications for weight loss alone (Pub. L. No. 108–173). Thus, leading to a scenario where a person’s income is a major factor in ease of access for these drugs.

Moreover, because discontinuation of GLP-1RAs often leads to weight regain, these medications must be regarded as a chronic therapy to sustain weight loss and prevent associated negative health outcomes. Meta-analyses have shown that the amount of weight regained after stopping treatment is typically proportional to the initial weight loss, and lifestyle interventions become increasingly difficult to maintain once the medication is discontinued (Berg et al., 2025). This highlights another significant barrier, the necessity of ongoing, long-term therapy to preserve weight management. Unlike a permanent cure for obesity or diabetes, GLP-1RAs provide benefits that diminish rapidly once treatment ends. Consequently, maintaining the therapeutic effects of GLP-1R agonism requires a long-term dosing regimen, much like medications used for other chronic conditions, such as statins for cholesterol control, ACE inhibitors and beta-blockers for blood pressure, or warfarin for blood thinning.

## 10 Expanding the use of GLP-1RAs to treat substance use disorders

To date, only a limited number of clinical trials have evaluated the effectiveness of GLP-1RAs for substance use disorders, yet initial findings are encouraging. For example, in a laboratory-based self-administration study, semaglutide was associated with significant reductions in both alcohol consumption and craving compared to placebo. Participants receiving semaglutide demonstrated lower post-treatment drinking and craving scores, and while changes in overall drinking days were not observed, there was a progressive decline in heavy drinking days within the semaglutide group (Hendershot et al., 2025). Additionally, the same study reported a decrease in cigarette smoking among participants who were smokers, suggesting potential cross-substance benefits of GLP-1RAs in this population (Hendershot et al., 2025).

Complementing these findings, a separate trial assessing the efficacy of exenatide for alcohol use disorder also showed no significant difference in heavy drinking days overall. Nevertheless, exenatide was shown to dampen alcohol-related cue reactivity in the ventral striatum, as measured by fMRI, and was associated with lower dopamine transporter levels (Klausen et al., 2022). Notably, an exploratory analysis revealed that among participants with obesity (BMI > 30), exenatide significantly decreased both the frequency of heavy drinking days and total alcohol consumption (Klausen et al., 2022).

With regard to nicotine use disorder, a randomized trial evaluating dulaglutide for smoking cessation found that abstinence rates at 12 weeks did not differ significantly between the dulaglutide and placebo groups (Lengsfeld et al., 2023). However, dulaglutide demonstrated a notable benefit by preventing the typical weight gain associated with quitting smoking, and participants receiving dulaglutide also experienced a significant reduction in HbA1c levels (Lengsfeld et al., 2023).

As of 02/05/2026, no clinical trials have been completed testing the efficacy of GLP-1RAs (GLP-1 RAs) in treating opioid use disorder (OUD), cocaine use disorder, or other stimulant use disorders. However, research in these areas is actively underway. At least two clinical trials are currently ongoing for OUD, investigating both liraglutide (Freet et al., 2024) and semaglutide (Freet et al., 2025). For cocaine use disorder, an ongoing clinical trial is registered (NCT07227948), and there is also an ongoing trial for methamphetamine use disorder (NCT07204249). These studies represent emerging efforts to evaluate the potential of GLP-1 RAs as pharmacological interventions for substance use disorders beyond their established role in obesity management. Together, these findings highlight the early but promising role of GLP-1 therapies in addressing both obesity and maladaptive reward-driven behaviors, as the clinical landscape continues to evolve.

## 11 Conclusion

GLP-1RAs have emerged as transformative agents in the landscape of obesity and T2DM management, offering glycemic control and robust reductions in food intake and body weight through an orchestrated interplay of central and peripheral mechanisms. By engaging GLP-1 Rs across the gut-brain axis, these agents potentiate physiological satiety signals, slowing gastric emptying, suppressing hunger cues, and diminishing the hedonic appeal of calorie-dense foods. This unified mechanism underpins the clinical efficacy observed across all FDA-approved GLP-1 RAs, though individual agents differ in pharmacokinetics and the relative emphasis on gastric versus central pathways. Short-acting agents, such as exenatide and lixisenatide, exert pronounced meal-time fullness primarily through delayed gastric emptying, while long-acting formulations including liraglutide, dulaglutide, and semaglutide sustain a more persistent satiety tone via direct activation of brain receptors.

The evolving public perception of obesity stigma reflects a gradual shift toward recognizing obesity as a complex medical condition influenced by biological and environmental factors, rather than a simple matter of personal responsibility. The increasing effectiveness and adoption of GLP-1RAs for obesity management are catalyzing a paradigm shift in both public and professional attitudes toward obesity. As pharmacological interventions demonstrate robust efficacy, obesity is increasingly recognized as a medical condition warranting comprehensive treatment, akin to other chronic diseases. However, societal and clinical perspectives remain divided. Recent survey data reveal that a substantial proportion of the public continues to attribute excess weight to personal responsibility, while a growing segment supports disease classification for obesity. Notably, clinician consensus on obesity as a chronic disease remains limited, with less than half routinely documenting it as such. This evolving landscape is further complicated by the body positivity movement, which challenges traditional pathologizing frameworks and advocates for a broader appreciation of body diversity and health beyond BMI. The advent of effective pharmacotherapies, such as GLP-1RAs, has disrupted these narratives, reframing obesity treatment within a medical context. Importantly, individuals expressing greater body dissatisfaction and weight-related concerns appear most interested in pharmacologic treatment, and demand for these agents extends beyond those with medical indications. Together, these developments underscore the dynamic interplay between medical advances, social attitudes, and the lived experiences of individuals with obesity, highlighting the need for continued dialog around the ethical, cultural, and clinical implications of obesity treatment.

Although GLP-1RAs have shown remarkable efficacy in weight management, several barriers limit their full integration into mainstream obesity care. Access and affordability are significant challenges, as are governmental policies, such as the current prohibition on Medicare coverage for weight loss medications in the US, restrict availability for many individuals who could benefit from these therapies. Furthermore, enduring weight-related stigma, skepticism about the long-term safety and effectiveness of these agents, and concerns about the potential for off-label or inappropriate use present additional obstacles. Addressing these challenges will require not only continued research and policy advocacy, but also culturally sensitive communication strategies to promote understanding, acceptance, and equitable access to GLP-1–based treatments.

The effects of GLP-1 therapies extend well beyond pharmacological appetite suppression. As reflected in the evolving strategies of food manufacturers and the food service industry, there is a growing recognition of the need to adapt product offerings to support individuals experiencing medication-induced appetite suppression. Portion-controlled, protein- and fiber-rich options are increasingly marketed not as restrictive diet foods, but as tools to help individuals maintain nutritional adequacy within smaller eating windows. This industry response underscores the broader societal shift toward viewing obesity through a medicalized lens, one that acknowledges biological drivers of eating behavior and the legitimacy of pharmacological interventions.

Importantly, research is now exploring the expansion of GLP-1RAs into the treatment of substance use disorders, including alcohol and nicotine dependence. Early clinical trials have shown promising results, with agents like semaglutide and exenatide demonstrating reductions in alcohol consumption and craving, as well as potential benefits for smoking cessation, such as reduced post-cessation weight gain. While further studies are underway to assess their efficacy for opioid, cocaine, and stimulant use disorders, current findings suggest that GLP-1 RAs may offer new avenues for addressing maladaptive reward-driven behaviors beyond obesity.

In sum, GLP-1RAs have catalyzed a paradigm shift in obesity treatment, facilitating meaningful weight loss, fostering healthier dietary patterns, and prompting industry-wide adaptation. Yet, their ultimate impact will be shaped not only by biological efficacy, but also by the evolving societal and professional discourse surrounding obesity, the medicalization of weight, and the persistent challenge of stigma. As research advances and real-world experience accumulates, the integration of these agents into multifaceted, patient-centered care models holds promise for reshaping the future of weight management.

## Footnotes

1
https://www.goodnes.com/vital-pursuit/

2
https://www.smoothieking.com/landing-pages/glp-1-smoothies
